# Appropriate indication and procedure for random skin biopsy in the diagnosis of intravascular large B‐cell lymphoma

**DOI:** 10.1111/ajd.13487

**Published:** 2020-12-18

**Authors:** Mayuko Sumi‐Mizuno, Atsushi Fukunaga, Hiroshi Kosaka, Yukihiro Imai, Tohru Nagano

**Affiliations:** ^1^ Department of Dermatology Kobe City Medical Centre General Hospital Kobe Japan; ^2^ Department of Dermatology Kobe University Graduate School of Medicine Kobe Japan; ^3^ Department of Clinical Pathology Kobe City Medical Centre General Hospital Kobe Japan

Dear Editor,

Intravascular large B‐cell lymphoma (IVLBCL) is a very rare subtype of extranodal malignant B‐cell lymphoma.^1^, ^2^ A useful method to diagnose IVLBCL is random skin biopsy, taken from healthy‐appearing skin.^3^ However, no consensus exists regarding random skin biopsy methods, and there is insufficient data regarding its accuracy.^4^ Thus, we aimed to determine potential correlations between positive diagnoses and biopsy techniques, serum lactate dehydrogenase (LDH) levels, as well as serum‐soluble interleukin 2 receptor (sIL‐2R) levels.

The study retrospectively reviewed 69 patients (43 males, average age: 65.6 ± 6.3 years, range: 21–87 years) who visited our department between April 2013 and March 2018 for random skin biopsy.

Clinically normal samples were obtained from at least three separate areas under local anaesthesia: incisional excision in 45 patients, punch biopsy in 24 cases. Biopsy specimens were stained with anti‐CD20 and CD79a antibodies.

A potential association between positive diagnoses and biopsy techniques was evaluated using Fisher Exact Test. Plotting the data showed that neither serum LDH nor sIL‐2R levels were normally distributed. Therefore, between‐group differences were determined using the Mann–Whitney *U* test. All analyses were conducted in SPSS (IBM, Japan). Significance was set at *P* < 0.05.

We diagnosed 10 patients with IVLBCL; the remaining 59 tested negative. One out of 10 IVLBCL‐positive cases was detected using punch biopsy, and incisional biopsy detected the rest. Fisher exact test did not detect a significant difference between biopsy techniques.

**Figure 1:**
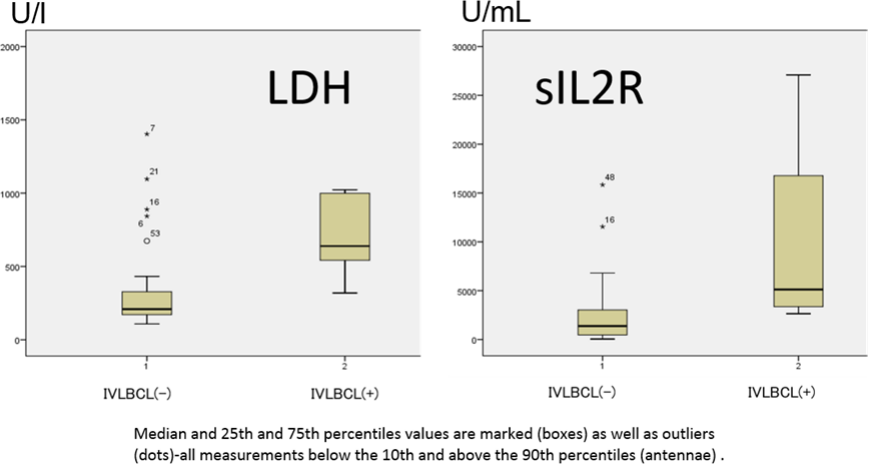
Box plot graphs of serum LDH levels and sIL‐2R levels of IVLBCL negative and positive patients.

Patients with IVLBCL had significantly higher (*P* < 0.01) serum LDH values (median = 639 U/L, range: 319–2962 U/L) than patients without IVLBCL (median = 208 U/L, range: 112–8408 U/L). Serum sIL‐2R levels were highly variable, but again, we observed a clear difference (*P* < 0.01) between patients with (median = 5120 U/mL, range 2646‐27 093 U/mL) and without IVLBCL (median = 1373 U/mL, range: <50‐15 833 U/mL) (Fig. 1).

We did not find significant differences in diagnostic accuracy among biopsy techniques, probably due to the small sample size. However, previous reports recommended against using punch biopsy as less tissue is available and results may not be representative.^4^ Incisional biopsy from more than three separate areas, on the other hand, provides more fat tissue and capillaries, and is recommended to avoid false negatives.

Random skin biopsies are usually performed by dermatologists at our hospital. The procedure was mostly used for investigating unknown fevers, pancytopenia and cerebral infarction. For patients with particularly poor health or haematological disorders, we sometimes hesitated to perform incisional random skin biopsies due to possible postoperative haematoma in fat tissues. A previous study indicated five factors (low O_2_ saturation, platelet count, serum LDH, serum sIL2R and unexplained fever) that helped to determine the indication for random skin biopsy in patients suspected of having IVLBCL.^2^, ^5^ In this study, we focused on serum LDH and sIL‐2R because high fever, low O_2_ saturation and low platelet count have low specificity, being often due to other complications.

In order to avoid false negatives in cases with serum sIL‐2R >2000 U/mL, we recommend incisional biopsy from at least three separate sites. Because in this study, serum sIL‐2R levels were over 2000 U/mL for all IVLBCL‐positive patients, whereas only 18 out of 59 IVLBCL negative patients were over 2000 U/mL. Conversely, when patients have sIL‐2R <500 U/mL and normal serum LDH levels, we do not recommend random skin biopsy because these patients had no lymphoproliferative disorders in our study. Moreover, in cases where patients experience bleeding and coagulation abnormalities, random skin biopsy should be postponed to prevent unexpected serious bleeding.
